# A novel recombinant variant of latent membrane protein 1 from Epstein Barr virus in Argentina denotes phylogeographical association

**DOI:** 10.1371/journal.pone.0174221

**Published:** 2017-03-22

**Authors:** Magdalena Gantuz, Mario Alejandro Lorenzetti, Paola Andrea Chabay, María Victoria Preciado

**Affiliations:** Instituto Multidisciplinario de Investigaciones en Patologías Pediátricas (IMIPP), CONICET-GCBA, Laboratorio de Biología Molecular, División Patología, Hospital de Niños Ricardo Gutiérrez, Buenos Aires, Argentina; Southern Illinois University School of Medicine, UNITED STATES

## Abstract

Epstein Barr virus (EBV) infection in Argentina occurs at an early age and occasionally develops infectious mononucleosis (IM). EBV is also related with lymphomas. LMP1, the viral oncoprotein is polymorphic and is used to define viral variants.

**Aim:**

To study LMP1 variants distribution among children with EBV+ malignant and benign conditions as well as in healthy carriers.

**Methods:**

Oral secretions and blood cells from 31 children with IM, and biopsies from 14 EBV+ reactive lymphoid hyperplasia and 33 EBV+ lymphomas were included. LMP1 was amplified by nested PCR and sequenced. Phylogenetic reconstructions were made under Maximun Likelihood, Bayesian and coalescent algorithms.

**Results:**

Six clades were defined (China1, China2, Med-, Alaskan, B95.8 and Argentine). Argentine variants, the most prevalent (46%), harbored 3 distinctive mutations and were a recombination between Raji and China1. Despite no pathology or compartment associations were observed for LMP1, the Argentine clade showed a phylogeographic association with our region. LMP1 estimated evolution rate was 8.591x10^-5^s/s/y and the estimated tMRCA for Raji and Argentine was 136ybp.

**Conclusions:**

An LMP1 Argentine clade was defined. LMP1 evolutionary rate was higher than expected for herpesviruses. The tMRCA for Raji and the Argentine agrees with African immigration and could explain the recombinant nature of the Argentine variant.

## Introduction

The Epstein Barr Virus (EBV), a member of the *Herpesviridae* family, was the first gammaherpervirus to be identified [1] and also the first human virus assumed to be oncogenic [2]. EBV is a ubiquitous virus that infects over 90% of the world’s population under two distinct epidemiological patterns. In developing regions, primary infection most commonly occurs during childhood, as the case of Argentina. Under this scenario, primary EBV infection takes place a few years after birth and seroconversion is almost universal by the age of 6 years. Primary EBV infection is usually asymptomatic but, in some cases, may develop into a mild case of infectious mononucleosis (IM) [3,4]. On the other hand, in developed regions or high socio-economic groups, less than 40% of the population becomes infected with EBV during childhood; however when primary infection is delayed until adolescence or early adulthood, it usually develops into a more severe case of IM [5]. EBV is also related to various tumors in humans, including Hodgkin lymphoma (HL), Burkitt lymphoma (BL), nasopharyngeal carcinoma (NPC), gastric carcinoma, T-cell lymphoma and lymphoproliferative disorders in immunocompromised individuals (PTLD).

After primary infection, EBV establishes a life-long latent infection in peripheral blood B-lymphocytes. During periodical reactivations, EBV is sheded into saliva at a low but continuous level. During latency, viral antigens are mostly down-regulated in order to avoid immune surveillance; in this context different viral latency programs are expressed under particular stimuli, related to the differentiation stage of the B cell. These differential latent antigen expression programs are also seen in EBV-associated diseases, and are defined according to the specific pattern of latent proteins expression.

Among latency proteins, EBV latent membrane protein 1 (LMP1) exhibits properties of a classical oncoprotein, since it induces both cell growth and inhibition of apoptosis in a variety of cell types *in vitro* [6]. Furthermore, it has also been demonstrated that LMP1 contributes to B cell and epithelial cell tumorigenesis *in vivo* in transgenic mice [7,8]. LMP1 is a 386 amino acid (~62kDa) integral membrane protein comprising a short N-terminal (N-ter) domain (amino acids 1 to 23), six transmembrane (TM) hydrophobic segments (amino acids24 to 186), and a long carboxy-terminal (C-ter) domain (amino acids 187 to 386). The N-ter region is required to ensure the correct orientation of LMP1 within the cellular membrane [9]. The TM domain is necessary for LMP1-LMP1 interaction, which enables protein oligomerization and localization into lipid rafts, and is also involved in protein signaling [10–13]. The C-ter region mediates LMP1 signals through the binding of adaptor molecules, including the TNF-receptor–associated factors (TRAFs) to the C-ter activations domain 1, 2, 3 (CTAR1, CTAR2, CTAR3). This interaction allows LMP1 to mimic a constitutively activated CD40 molecule, a cellular receptor that belongs to the TNF super family, in a ligand-independent manner. In consequence, LMP1 stimulates multiple cellular signaling pathways that in turn activate NF-κB, AP-1, inhibitor-of-differentiation 2 and 3 (Id2 and Id3), and STAT-mediated transcription [14].

Comparative genomic sequencing of herpesvirus isolates is revealing novel aspects of recent evolution, where recombination among strains has emerged as a general phenomenon. Also, certain latency genes from gammaherpesviruses show signs of widespread diversifying selection [15]. In particular, LMP1 displays more nucleotide diversity than other viral genes and, like other EBV latent genes such as EBNA-1, -3A, -3B, -3C, -LP and LMP2A, it seems to have experienced an accelerated protein evolution due to positive selection [16,17]. Furthermore, intra-host genetic variability of LMP1 observed in EBV-infected adults was comparable to whole genome variability seen in early infection with RNA viruses such as West Nile Virus and HIV-1 [18].

Previous reports described EBV genetic variability related to different EBV associated diseases and/or specific geographic locations [19]. In this context, concerning the analysis of the three different LMP1 regions, the C-ter was the most extensively studied [20]. Based solely on the latter, a classification scheme that categorizes variants into classes that consider signature amino acid changes relative to the prototypic B95.8 LMP-1 was developed [21]. Under this scheme, the variants are named according to the geographic region where they were first isolated: Alaskan, China 1, China 2, China 3, Mediterranean+ (Med+), Med−, and North Carolina (NC) (21). Since only few studies comprised LMP1 entire gene sequence, it is unknown whether this classification scheme could still be applicable or new approaches need to be developed to discuss the geographical distribution and/or the pathogenic role of LMP1 [22,23].

Given that the incidence of EBV-associated diseases differs among geographical regions and that EBV genetic variants also differ through these geographic zones, the characterization of those structural changes in LMP1 variants specifically associated to tumors is still a matter of interest and debate.

The vast majority of papers reporting sequence variation on LMP1 gene have been performed in adult patients but only a few considered pediatric samples. In the present study, variations in LMP1 gene complete sequence from pediatric isolates with benign (IM) and malignant EBV related lymphomas, as well as from pediatric healthy carriers were characterized and LMP1 evolution was inferred.

## Materials and methods

Hospital’s ethic committee reviewed and approved this study, which is in accordance with the human experimentation guidelines of our institution and with the Helsinki Declaration of 1975, as revised in 1983. A written informed consent was obtained from all patient’s parents or tutors. The Review Board of our institution is: “Comité de Ética en Investigación del Hospital de Niños Ricardo Gutiérrez”.

### Patients and samples

A total of 78 pediatric patients were enrolled: i) 31 cases were confirmed EBV+ IM, median age of 4.5 years (age range 1.5 to 17 years), 52% males, ii) 33 patients were diagnosed with EBV-associated lymphoma (T) [(26 HL and 7 Non-HL(NHL)], median age 9 years (age range 4 to 18 years), 70% males and iii) 14 cases of EBV+ reactive lymphoid hyperplasia (RLH), median age 8 years (age range, 3 to 16 years), 57% males (S1 Table). Peripheral blood (6 ml) and oral secretion (OS) samples were obtained from patients with presumptive acute IM at the time of diagnosis. Lymph node biopsies from presumptive lymphomas and RLH were collected for diagnosis. Lymphomas were treatment naïve. The biopsies were sectioned; one half was formalin-fixed and paraffin embedded and the other half was conserved at -80°C. Lymphoma diagnosis and histological classification, as well as RLH diagnosis were assessed at the Pathology Division.

### Serological assays

IM was identified on clinical grounds and confirmed by an indirect immunofluorescent assay (IFA) for EBV-VCA antigen on substrate slides (MBL, Woburn, MA, USA). Patients with IgM antibodies against EBV-VCA were included in the study. As a differential diagnosis for other mononucleosis-like conditions, IgM anti-Cytomegalovirus and anti-*Toxoplasma gondii* were assessed by ELISA. All patients were negative for both of them. All patients were HIV negative as well.

### EBERs in situ hybridization

EBV presence was assessed on formalin-fixed, paraffin embedded lymph node biopsy sections (lymphoma patients) and resected tonsils (RLH patients) by means of a commercial *in situ* hybridization (ISH) kit for EBERs according to the manufacturer’s instructions (Dako, Carpinteria, CA, USA). In lymphoma cases, those with positive nuclear staining in tumor cells without staining in infiltrating lymphocytes were included. Tonsils with positive nuclear staining in lymphocytes were selected.

### DNA extraction and amplification

Peripheral blood mononuclear cells (PBMC) were separated from whole blood with Ficoll-Paque plus (GE Healthcare, Little Chalfont, UK). Genomic DNA was extracted from PBMC, OS and fresh lymph node and tonsil biopsies using QIAamp DNA Mini Kit (QIAGEN, Hilden, Germany) following manufacturer’s instructions. EBV type was assessed by amplification of the EBNA3C gene as previously described [24]. The complete coding sequence of LMP1, 1390pb, was amplified by nested PCR. Primers used in the first round were LMP11 5' TGATTAGCTAAGGCATTCCCA 3' (B95.8 prototype EBV genome, GenBank accessions Nº V01555.2, coordinates 168075–95) and LMPEco 5' CCGTACTGCCTCCGGCAGAC 3' (169607–27) and in the second round A2 5' GCCTATGACATGGTAATGCCTA 3' (168124–45) and E2 5' CTTTCCTCAACTGCCTTGCT 3' (168124–45). PCR amplicons (1390bp, with variations according to structural polymorphisms) was separated by electrophoresis in 2% agarose gel, stained with ethidium bromide and visualized under UV light. The specific amplification product was recovered and purified with QIAEXII gel extraction kit (QIAGEN, Hilden, Germany) according to manufacturer’s instructions. These purified PCR products were directly sequenced using Big Dye Terminator v3.1 kit (Applied Biosystems, Foster City, CA, USA) in an automated Genetic Analyzer 3130xl (Applied Biosystems, Foster City, CA, USA) in four segments using a contig strategy with primer A2, E2, B 5`ATTGTCAGGACCACCTCCAG3' (168568–88) and C 5' TTCCTTCTCTAACGCACTTTCTC 3' (169141–63). Primers were designed within viral conserved regions and were tested in EBV+ cell lines (B95.8, Raji and P3HR1) and in an EBV- cell line (Ramos).

### Sequence analysis

Sequences were aligned and edited with Bioedit software [25]. For phylogenetic analysis, the most appropriate model of evolution for this region was inferred using jModelTest [26] according to the Akaike Information Criterion (AIC) **(**S2 Table**)**.

#### a) Phylogenetic analysis

Maximum likelihood (ML) trees: The ML tree was estimated using the previously defined evolutionary model and bootstrapping was performed after 1000 replicates under the ML substitution model. Full length LMP1 reference sequences from EBV+ cell lines B95.8 (**V01555.2**), GD1 (**AY961628.3**), GD2 (**HQ020558.1**), Akata (**KC207813.1**), Cao (**X58140.1**), Raji (**KF717093.1**), AG876 (**DQ279927.1**) and Mutu (**KC207814.1**) and from isolates related to specific geographical regions previously described for C-ter classification, namely China 1 (**AY337723.1**), China 2 (**AY337724.1**), Med+ (**AY337722.2**), Med- (**AY337721.2**), NC (**AY337726.2**), Alaskan (**AY337725.1**), HKNPC1 (**JQ009376.2**), M81 (**KF373730.1**) were downloaded from GenBank. The whole phylogenetic analysis were performed using PhyML 3.0 [27] and the graphical representation and edition of the phylogenetic tree was performed with FigTree (version 1.3.1, http://tree.bio.ed.ac.uk/software/figtree/).

#### b) Recombination analysis

Presumptive recombinant sequences were analyzed with Simplot software [28] under neighbor-joining algorithms with a 200bp fragment length (window), 20bp passes and 100 repetitions.

#### c) Phylogeographic analysis

The degree of association between geographic origin and position of the sequence in the virus phylogeny was estimated using the program BaTS (Bayesian Tip-Significance testing); the Parsimony Score [PS], Association Index [AI] and the Monophyletic Clade Size [MC] statistics were determined (S3 Table). The BaTS program examines a posterior sample of trees generated by a Bayesian Markov Chain Monte Carlo (MCMC) approach implemented in BEAST (Bayesian Evolutionary Analysis Sampling Trees). The BaTS program was performed with 1,000 replications, p<0.05 was considered significant.

#### d) Co-estimate of substitution rate and the time to most recent common ancestor (tMRCA)

A Bayesian coalescent analysis was carried out on a set of 68 sequences from samples with an available sampling time with Beast2 [29]. Sequence analysis programs such as Bayesian Evolutionary Analysis by Sampling Trees (BEAST) utilize the genetic variation present in a sample to simultaneously estimate both demographic and evolutionary parameters in the context of time and space [30–33]. A minimum of two independent MCMC simulations for each model-clock combination were performed for no less than 50 million generations. Genealogies were estimated under: 1) strict molecular clock (Strict), 2) relaxed molecular clock with an “uncorrelated log-normal model” (UCLN) distribution of rates. The parameters were estimated under three different demographic models: hypothesis of a constant population size, coalescent exponential population and Bayesian skyline coalescent model under the general time-reversible (GTR) model of base substitution (S4 Table). After confirming MCMC convergence and congruence among parameter estimates from different prior sets, summary trees were created using TreeAnnotator [29] and edited in FigTree software.

### Statistical analysis

Statistical analysis to infer the association between both LMP1 variants and polymorphisms with the different pathological conditions was performed using Graph-Pad InStat software. For the univariate analysis, Chi-square or Fisher’s exact test were used to assess the association between categorical variables. All tests were two sided, and a p value of less than 0.05 was considered statistically significant.

## Results

Seventy eight pediatric patients were included in this study, 31 EBV+ IM cases, 33 EBV associated lymphomas [Tumors, (T)], and 14 EBV+ reactive lymphoid hyperplasia (RLH), as representatives of benign EBV primary infection, malignant EBV infection and normal healthy carriers, respectively. EBV association was properly confirmed by specific serological tests for IM cases and by EBERs in situ hybridization assay for the biopsy specimens. After PCR amplification and direct sequencing of the amplicons, full length LMP1 sequences could be assembled for 25/31 IM patients, 26/33 lymphoma cases and for 14/14 RLH cases. A total of 90 full length LMP1 sequences, considering both PBMC and OS compartments in IM cases, were analyzed. Unfortunately, due to the institution’s ethic committee guidelines, PBMC and OS samples were not available from child`s T and RLH. Only one LMP1 variant was detected in each sample by means of nested PCR followed by direct sequencing, but the presence of other underrepresented LMP1 variants cannot be ruled out. Additionally, there were no differences concerning variant distribution between both anatomical compartments (PBMC and OS) in each IM patient; so only one sequence per IM case was included in the following analysis.

### Polymorphisms characterization

Concerning the N-ter region of LMP1, which has been studied to a lesser extent than the C-ter region, the most studied polymorphism comprehends the loss of an XhoI (CTCGAG) restriction site [34,35]. In our series, mutations in XhoI site were detected in 4/25 (16%) IM, 7/26 (27%) T and 1/14 (7%) RLH (Table 1) and no association between this polymorphism and malignancy was observed (p>0.05). Concerning the TM domain, in a previous report, two polymorphisms were identified within the TM domain in HIV patients as markers of increased NF-κB activity in vitro, namely I124V/I152L and F144I/D150A/L151I [36]. In our series I124V/I152L was detected in 9/25 (36%) IM, 15/26 (58%) T and 6/14 (43%) RLH. Meanwhile, F144I/D150A/L151I was detected in 2/25 (8%) IM, 3/26 (12%) T but was absent in RLH (Table 1). None of these polymorphisms was associated with a particular pathology (p>0.05 in each case). On the other hand, several signature hotspot mutations, along with a 30bp deletion (del30), a 15bp insertion (ins15) encoding a Janus Kinase 3 (JAK3) signaling motif and a variable numbers of 33bp tandem repeats (rep33), define LMP1 variants at the C-ter region, some of which have been described to alter LMP1 signaling activity [21,37]. The occurrence of del30 in IM was 13/25 (52%), 18/26 (69%) in T, and 7/14 (50%) among RLH. The ins15 was detected either within the third or the fourth repetitive unit, and was observed in 12/25 (48%) IM, 15/26 (57%) T, and 7/14 (50%) RLH. Finally, the number of rep33 ranged between 3 ½ and 8½ units, where 5½ repeats was the most frequently detected polymorphism in 13/25 (52%) IM, 21/26 (81%) tumor samples, and 6/14 RLH (42%) (Table 1). Only the presence of 5 ½ rep33 was significantly associated with tumors (p = 0.039 vs. IM and p = 0.0312 vs. RLH, Fisher´s exact test)

**Table 1:** Polymorphisms and variant characterization.

	**LMP1 N-ter**	**LMP1 TM**			**LMP1 C-ter**			**Full length**
**Isolate**	**Xho1 ctcgag**	**position 169055 (CxT)**	**I124V/I152L**	**F144I/D150A/L151I**	**del30**	**rep33 and ins15**	**Variant**	**Variant**
IM1	wt	wt	wt	F144I/D150A/L151I	+	5 ½	China1	China1
IM2	wt	wt	wt	wt/D150G/L151I	wt	4 ½	China2	Med-
IM6	wt	+	I124V/I152L	wt/wt/L151I	+	5 ½ + I (4°)	China1*	V-Arg
IM7	wt	+	I124V/I152L	wt/wt/L151I	+	5 ½ + I (3°)	China1*	V-Arg
IM8	wt	+	I124V/I152L	wt/wt/L151I	+	5 ½ + I (3°)	China1*	V-Arg
IM9	wt	wt	wt	wt/wt/wt	+	6 ½	Med-	Med-
IM10	wt	wt	wt	wt/wt/wt	wt	6 ½	Med-	Med-
IM11	wt	wt	wt	wt/wt/wt	wt	5 ½	Med-	Med-
IM12	wt	+	I124V/I152L	wt/wt/L151I	+	5 ½ + I (4°)	China1*	V-Arg
IM13	wt	wt	wt	wt/wt/wt	wt	5 ½	Med-	Med-
IM16	wt	+	I124V/I152L	wt/wt/L151I	+	5 ½ + I (4°)	China1*	V-Arg
IM17	ctctag	wt	wt	F144I /wt/wt	wt	4 ½	China2	China2
IM18	wt	wt	wt	wt/wt/wt	+	4 ½ + I (3°)	B95.8	B95.8
IM19	wt	+	I124V/I152L	wt/wt/L151I	+	4 ½ + I (3°)	China1*	V-Arg
IM20	wt	wt	wt	wt/wt/wt	wt	4 ½ + I (3°)	B95.8	B95.8
IM21	wt	+	I124V/I152L	wt/wt/L151I	+	5 ½ + I (4°)	China1*	V-Arg
IM22	wt	wt	wt	wt/wt/wt	wt	5 ½	Med-	Med-
IM23	ctctgg	wt	wt	F144I/wt/L151I	wt	5 ½	China2	China2
IM24	ctctag	wt	wt	F144I/wt/L151I	wt	3 ½	China2	China2
IM26	wt	wt	wt	wt/wt/wt	+	5 ½	China1	Med-
IM27	wt	wt	wt	F144I/D150A/L151I	+	3 ½	China1	China1
IM28	ctctag	wt	wt	F144I/wt/L151I	wt	4 ½	China2	China2
IM29	wt	wt	wt	wt/wt/L151I	wt	4 ½ + I (3°)	B95.8	B95.8
IM30	wt	+	I124V/I152L	wt/wt/wt	+	5 ½ + I (4°)	China1*	V-Arg
IM31	wt	+	I124V/I152L	wt/wt/L151I	wt	4 ½ + I (3°)	Raji	V-Arg
	**LMP1 N-ter**	**LMP1 TM**			**LMP1 C-ter**			**Full length**
**Isolate**	**Xho1 ctcgag**	**position 169055 (CxT)**	**I124V/I152L**	**F144I/D150A/L151I**	**del30**	**rep33 and ins15**	**Variant**	**Variant**
T1	wt	+	I124V/I152L	wt/wt/L151I	+	5 ½ + I (4°)	China1*	V-Arg
T2	wt	wt	wt/wt	F144I/D150A/L151I	+	5 ½	China1	China1
T3	ctctag	wt	wt/wt	F144I/wt/L151I	wt	5 ½	Alaskan	Alaskan
T4	ctctgg	wt	I124T/wt	F144I/wt/L151I	wt	3 ½	Alaskan	Alaskan
T5	wt	+	I124V/I152L	wt/wt/L151I	+	5 ½ + I (4°)	China1*	V-Arg
T6	wt	+	I124V/I152L	wt/wt/L151I	+	6 ½ + I (4°)	China1*	V-Arg
T8	ctttag	wt	wt/wt	F144I/wt/L151I	wt	3 ½	Alaskan	Alaskan
T9	wt	+	I124V/I152L	wt/wt/L151I	+	5 ½ + I (4°)	China1*	V-Arg
T10	wt	+	I124V/I152L	wt/wt/L151I	+	5 ½ + I (4°)	China1*	V-Arg
T11	wt	wt	wt/wt	F144I/D150A/L151I	+	5 ½	China1	China1
T14	wt	+	I124V/I152L	wt/wt/L151I	+	5 ½ + I (4°)	China1*	V-Arg
T15	ctctag	wt	wt/wt	F144I/wt/L151I	wt	5 ½	Alaskan	Alaskan
T16	ctctag	wt	wt/wt	F144I/D150A/L151I	+	5 ½	China1	China1
T18	wt	wt	wt/wt	wt/wt/wt	wt	5 ½	Med-	Med-
T19	wt	+	I124V/I152L	wt/wt/L151I	+	5 ½ + I (4°)	China1*	V-Arg
T23	wt	+	I124V/I152L	wt/wt/L151I	+	5 ½ + I (4°)	China1*	V-Arg
T24	wt	+	I124V/I152L	wt/wt/L151I	+	5 ½ + I (4°)	China1*	V-Arg
T25	wt	+	I124V/I152L	wt/wt/L151I	+	5 ½ + I (4°)	China1*	V-Arg
T26	wt	wt	wt/wt	wt/wt/wt	wt	4 ½ + I (3°)	B95.8	B95.8
T27	wt	+	I124V/I152L	wt/wt/L151I	+	5 ½ + I (4°)	China1*	V-Arg
T28	ctctag	wt	wt/wt	F144I/wt/L151I	wt	5 ½	Alaskan	Alaskan
T29	wt	+	I124V/I152L	wt/wt/L151I	+	5 ½ + I (4°)	China1*	V-Arg
T30	wt	+	I124V/I152L	wt/wt/L151I	+	6 ½	China1*	V-Arg
T31	ctctgg	wt	wt/wt	F144I/wt/L151I	wt	5 ½	China2	China2
T32	wt	+	I124V/I152L	wt/wt/L151I	+	5 ½ + I (4°)	China1*	V-Arg
T33	wt	+	I124V/I152L	wt/wt/L151I	+	5 ½ + I (4°)	China1*	V-Arg
	**LMP1 N-ter**	**LMP1 TM**			**LMP1 C-ter**			**Full length**
**Isolate**	**Xho1 ctcgag**	**position 169055 (CxT)**	**I124V/I152L**	**F144I/D150A/L151I**	**del30**	**rep33 and ins15**	**Variant**	**Variant**
RLH1	wt	+	I124V/I152L	wt/wt/L151I	+	5 ½ + I (4°)	China1*	V-Arg
RLH2	wt	+	I124V/I152L	wt/wt/L151I	+	5 ½ + I (4°)	China1*	V-Arg
RLH3	wt	wt	wt/wt	wt/wt/wt	wt	5 ½	Med-	Med-
RLH4	wt	+	I124V/I152L	wt/wt/L151I	+	5 ½ + I (4°)	China1*	V-Arg
RLH5	wt	+	I124V/I152L	wt/wt/L151I	+	4 ½ + I (3°)	China1*	V-Arg
RLH6	ctctag	wt	wt/wt	F144I/wt/L151I	wt	6 ½	Alaskan	Alaskan
RLH7	wt	wt	wt/wt	wt/wt/wt	wt	4 ½	Med-	Med-
RLH8	wt	wt	wt/wt	wt/wt/wt	+	4 ½ + I (3°)	B95.8	B95.8
RLH9	wt	+	I124V/I152L	wt/wt/L151I	+	6 ½	China1*	V-Arg
RLH10	wt	wt	wt/wt	wt/wt/wt	wt	4 ½ +I (4°)	B95.8	B95.8
RLH11	wt	wt	wt/wt	wt/wt/wt	wt	3 ½	Med-	Med-
RLH12	wt	wt	wt/wt	wt/wt/L151I	wt	8 ½	Med-	Med-
RLH13	wt	wt	wt/wt	wt/wt/wt	wt	5 ½	Med-	Med-
RLH14	wt	+	I124V/I152L	wt/wt/L151I	+	5 ½ + I (4°)	China1*	V-Arg

China 1* refers to China 1 related sequences but have additional substitutions.

### LMP1 variants characterization

**Fig 1:**
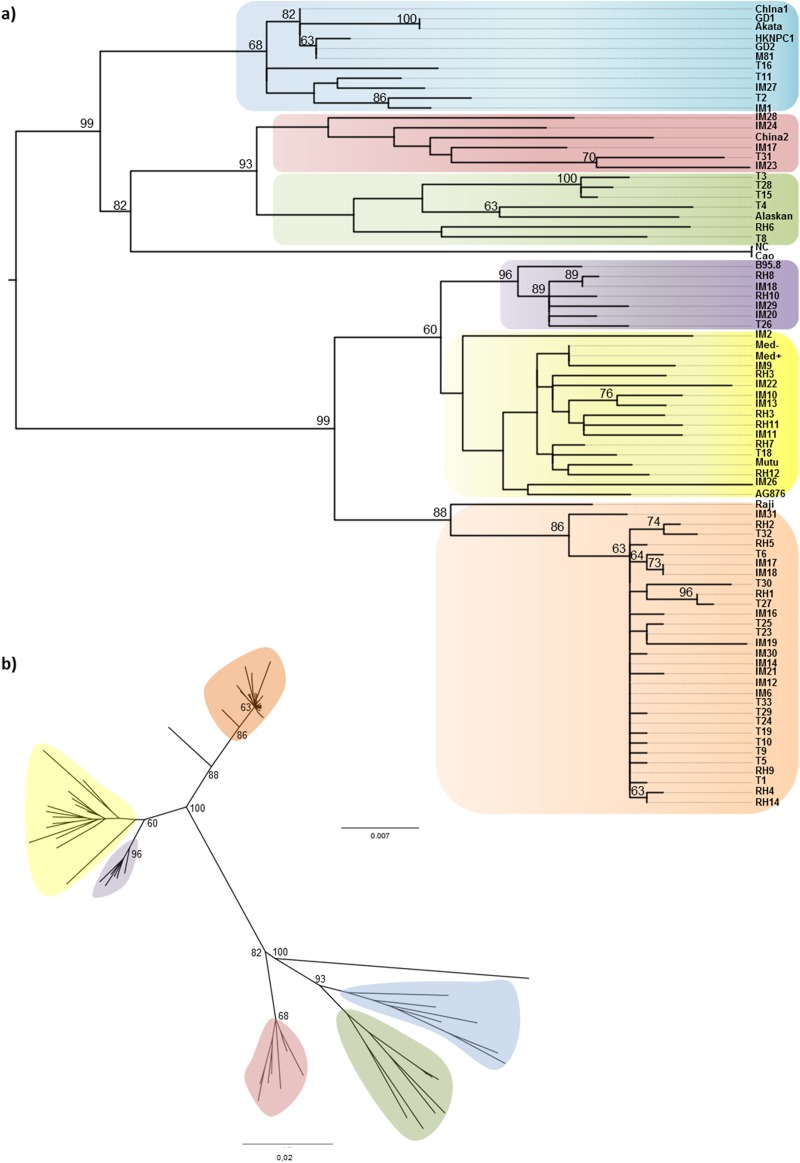
Phylogenetic reconstruction. Phylogenetic tree obtained from the alignment of full length LMP1 sequences amplified from patients with IM, EBV positive lymphomas (T) and healthy donors (RLH). GenBank downloaded reference sequences were included (B95.8, GD1, GD2, Akata, Cao, Raji, AG876, China 1, China 2, Med+/-, NC, and Alaskan, HKNPC1, M81). (A) The tree was rooted by middlepoint method, bootstrap values were obtained after 1000 resamplings, and only bootstrap values over 60% are shown. (B) Unrooted tree. Raji clade (orange), Mediterranean clade (yellow), B95.8 clade (purple), Alaskan clade (green), China2 clade (red) and China 1 clade (blue).

In order to examine genetic variability and evolutionary relationships, 65 LMP1 full length sequences, 25 IM, 26 T and 14 RLH, from pediatric isolates were aligned together with full length LMP1 sequences from EBV+ cell lines and from geographically-specific isolates obtained from GenBank. Phylogenetic reconstructions were inferred under maximum likelihood methods. LMP1 sequences included in our analysis clustered into six clades; four smaller ones related to China1, China2, Alaskan or B95.8 and to major clades, one related to Med+/Med- and a predominant clade related to Raji. Of notice, other than the reference Raji variant, this predominant clade only included isolates from our pediatric series, 9 IM, 15 T and 6 RLH (Fig 1A) which all contained three signature substitutions: C → T 169055 (intron), A → G 168951 (I124V in TM domain 4) and A → C 168867 (I152L in TM domain 5).

**Fig 2:**
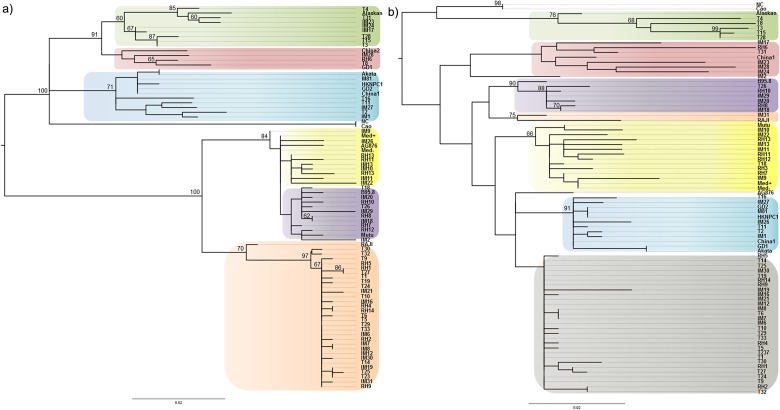
Phylogenetic reconstruction. (A) Phylogenetic tree obtained from the alignment of sequences from the N-ter and TM domain of LMP1 from patients with IM, EBV positive lymphomas (T) and healthy donors (RLH). GenBank downloaded reference sequences were included (B95.8, GD1, GD2, Akata, Cao, Raji, AG876, China 1, China 2, Med+/-, NC, and Alaskan, HKNPC1, M81). Raji clade (orange), Mediterranean clade (yellow), B95.8 clade (purple), Alaskan clade (green), China2 clade (red) and China 1 clade (blue). (B) Phylogenetic tree obtained from the alignment of sequences from the C-ter domain of LMP1. Raji clade (orange), Mediterranean clade (yellow), B95.8 clade (purple), Alaskan clade (green), China2 clade (red), China 1 clade (blue) and China1* clade (related to China1 (grey)). Bootstrap values were obtained after 1000 resampling, only bootstrap values over 60% are shown.

Strikingly, phylogenetic results were not in accordance with our previous analysis on LMP1 sequences when only the C-ter region was analyzed and where China1 related sequences were prevalent in our geographical region [38]. In order to test our previous results, phylogenetic reconstructions were inferred again but considering in the alignment only the N-ter and TM domains, and independently the C-ter domain (Fig 2). When analyzing the N-ter/TM phylogenetic tree, the topology of the predominant clade matched exactly with that of the full length LMP1 tree, clustering with Raji and significantly differing from the China1 clade (Fig 2A). On the other hand, the C-ter phylogenetic tree also displayed this predominant clade as a separate one, which included no reference sequence but didn’t differ significantly from the China1 clade either (bootstrap <60%). Hence, if only considering the C-ter region, this predominant clade could be considered as a China 1 related clade (China1*), in accordance with our previous observations (Fig 2B). When considering full length LMP1, the divergence of the China1* clade from the China1 clade is mainly due to the three signature polymorphisms (intron, TM4 and TM5) harbored by these isolates. In the same phylogenetic tree, Raji and the isolate IM31 clustered together in a separate clade. The latter fact suggested that this predominant clade could in fact be the result of a recombination process between Raji and China1 related variants. Since it was previously suggested that specific LMP1 variants could be associated with certain pathological conditions, we tested this hypothesis by comparing EBV+ tumor samples (HL and NHL) with EBV+ benign pathology (IM) and healthy carriers (RLH); however no statistical association was disclosed between any particular LMP1 variant and benign or malignant conditions (p>0.05 in each case).

### Recombination analysis

**Fig 3:**
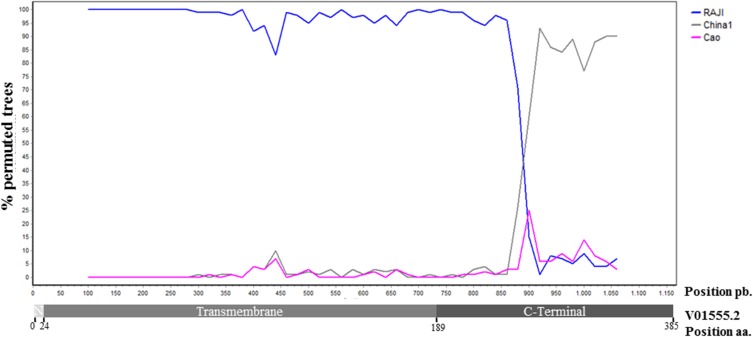
Recombination analysis. Bootscan analysis of the Argentine variant represented by sequence RLH14, against Raji (blue), China1 (grey) and Cao (pink) parental sequences. The rep33 region was omitted from the alignment since they presented no mutations within and since the parental sequences had different rep33 units each. Analyses parameters were: 200pb window, 20pb paces and 100 repetitions under neighbour-joining algorithm.

**Fig 4:**
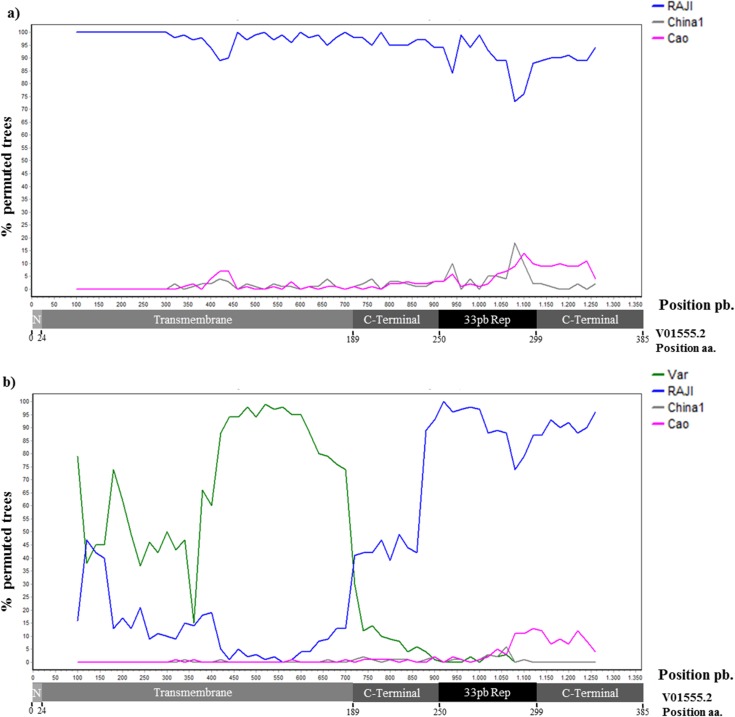
Recombination analysis. (A) Bootscan analysis of sequence from isolate IM31 against Raji (blue), China1 (grey) and Cao (pink) parental sequences. (B) Bootscan analysis of sequence from isolate IM31 against Argentine variant, represented by sequence RLH14 (green) and Raji (blue), China1 (grey) and Cao (pink) parental sequences. The rep33 region was omitted from the alignment since they presented no mutations within and since the parental sequences had different rep33 units each. Analyses parameters were: 200pb window, 20pb paces and 100 repetitions under neighbour-joining algorithm.

As previously suggested by our results, and to assess the possibility of a recombination process, each parental sequence (Raji and China1) was tested against a representative sequence of the predominant clade, RLH14, and Cao reference sequence was used as an outgroup. A recombination point was detected in the C-ter domain within the region comprising the 33 bp tandem repeats (Fig 3). This result indicates that the predominant clade detected in our LMP1 complete sequence phylogenetic analysis could indeed be the result of a recombination between Raji and China1 sequences. This can be deduced by the fact that almost 100% of the generated trees during the bootscaning process (% permuted trees) were similar to Raji reference sequence through the N-ter and TM domain but were similar to China1 sequence after the recombination point in the C-ter domain. We also tested IM31 isolate since this sequence clustered together with Raji both in the N-ter and C-ter, but contains C→T 169055, A→G 168951 (I124V) and A→C 168867 (I152L) mutations in the N-ter and TM domains, characteristic of the sequences in the predominant clade. Variant IM31 showed no recombination point when tested against Raji, China1 and Cao (Fig 4A), but when tested against HR14 (as a representative of the predominant clade) recombination points were observed in the N-ter and TM domain (Fig 4B). Since isolate IM31 is identical to Raji, but contains the mentioned mutations, these results suggest that IM31 could be a parental sequence for the predominant clade isolates.

### Phylogeographical analysis

Given that the sequences in the predominant clade described in Fig 1 represented 46% (30/65) of the pediatric isolates from our geographical region and were not described in other areas, we assessed the possibility that these sequences represent a distinctive LMP1 variant preferentially circulating in our region.

**Fig 5:**
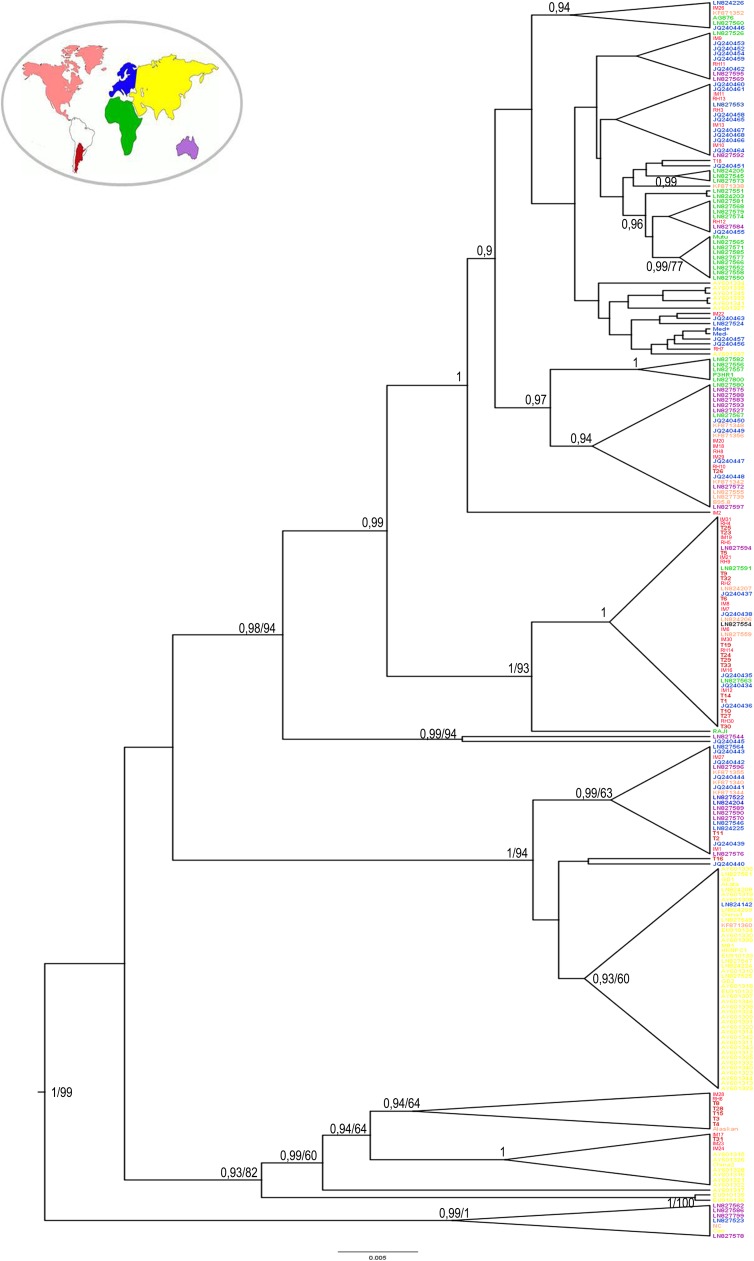
Phylogeographic reconstruction. Phylogenetic tree obtained from the alignment of the amplification fragment of LMP1 full length sequences from patients with IM, EBV positive lymphomas (T) and healthy donor (RLH) along isolates from distinct geographical regions reported in GeneBank. The variants were categorized according to their geographical origin as Asia (yellow), Europe (blue), Africa (green), North America (orange), Oceania (purple) and Argentina (red). Bayesian posterior probabilities over 0.85 and bootstrap over 60% are shown.

For phylogeographic analysis purposes, we retrieved from GenBank 177 full length LMP1 sequences from Asia (N = 59; 24.3%), Europe (N = 46; 18.9%), Africa (N = 33; 13.6%), North America (NA) (N = 17; 7%), Oceania (N = 22; 9%) and analyzed them along with the 65 sequences from our Argentinean pediatric isolates under maximum likelihood and Bayesian algorithms. As shown in Fig 5 those sequences in the predominant clade in Fig 1 still clustered together as a separate clade (posterior probability (pp = 1), which also included some sequences from NA, Africa and Europe. On the other hand, sequences from Asia, which included isolates from China, Vietnam and Japan also formed separate and independent clusters which included reference sequences China1 and China2 (pp = 0.99/63 and pp = 1, respectively). As well, African isolates segregated independently including reference sequences P3HR1 and Mutu (pp = 0.99/71 and pp = 1, respectively). European sequences formed 2 clusters which also included our pediatric isolates that previously clustered together with Med+/Med- reference sequences. In order to test for statistical significance of the phylogenetic reconstruction, a bayesian statistical association analysis (BaTS) was performed in BEAST. The Association Index (AI), significantly differed from that expected by chance (p<0.001). Monophyletic Clade index (MC), which denotes the association with the geographic origin of the clade, were statistically significant for Asian, African, NA, Oceania and the predominant clade from our pediatric series (p<0.001, p<0.001, p = 0.001, p = 0.002 and p = 0.004, respectively) (S2 Table).

Since the sequences in the predominant clade previously described in Fig 1 represents 46% (30/65) of the pediatric isolates from our geographical region, harbor 3 distinctive mutations, are a recombination between Raji and China1 variants, are evenly distributed among the studied population (IM, T and RLH) and have a monophyletic origin within our region, we decided to term them as the newly characterized Argentine variant.

### LMP1 molecular evolution

LMP1 has been shown to be the viral gene with the highest degree of variation and has depicted a high number of polymorphisms at the amino acid level in our pediatric series (S1 Fig). The number of variants that are defined by LMP1 sequence variation exceeds by far that of any other EBV gene. Thus, LMP1 gene provides the most suitable genetic locus for EBV variants identification, and it is as well the one which displays the highest sensitivity and specificity for molecular epidemiology studies on EBV infection [39].

As for the substitution rate calculated for LMP1, although this is not a longitudinal study, advances in computational algorithms have enabled the incorporation of temporal information from time-structured sequence datasets into strict or relaxed molecular clock models [30–32], which can then be used to estimate the timing of epidemiologically important events. Therefore, we used temporally structured sequence data to estimate the evolutionary rate of LMP1 gene. A dataset was constructed with the sequences described here and other complete LMP1 sequences with available sampling dates (1972–2013) from other geographic regions, which were retrieved from GenBank. The sampling time, spanning 41 years, was incorporated in a Bayesian Markov Chain Monte Carlo (MCMC) analysis using BEAST 2 [29] in order to determine the evolution rate and the tMRCA. Genealogies were estimated under: 1) strict molecular clock, 2) relaxed molecular clock with an uncorrelated lognormal (UCLN) distribution of rates. The parameters were estimated under three different demographic models: 1) hypothesis of a constant population size, 2) coalescent exponential population and 3) Bayesian skyline coalescent model. The Bayesian MCMC inference for each combination converged efficiently on a posterior mean value for all parameters, independently of the demographic model applied. The Bayes Factor (BF) significantly supported the UCLN clock model over the others (S4 Table). The mean evolutionary rate estimated under UCLN and constant population size was 8.591 x 10–5 subs/site/year [4.779–12.89 x 10–5 subs/site/year, 95% Highest Posterior Density (HPD)]. In particular, the tMRCA of Argentine and Raji clades was 136 ybp (76–223 ybp, 95% HPD).

To test and confirm the temporal signature observed in our data, we performed a tip-date randomization test. The sequence-sampling time associations were randomized 20 times, and the evolutionary rates were re-estimated. The hypothesis of significant temporal structure is rejected when the value of the mean evolutionary rate estimated from the real data fell within the 95% HPDs of those estimated from the randomized data, as previously described [40,41]. In our analysis, the hypothesis of temporal structure was accepted since the value of the mean real evolutionary rate did not fell within the 95% HPDs of the randomized data (S2 Fig).

## Discussion

In this work we aimed to study polymorphisms in LMP1 from pediatric samples because of the particular incidence of Hodgkin lymphoma (HL) in our geographic region, where our group has previously reported a high incidence in children below ten years of age in contrast to what is described in the United States and most European countries where HL shows an incidence peak between 15 and 30 years. Moreover, EBV was associated with 31% of adult HL cases, but rose up to 54% in pediatric patients. Furthermore, EBV + cases had a median age of 8 years, versus a median of 12 years observed in EBV- cases [3]. In parallel, we also described in a Burkitt lymphoma series from our country, an EBV association specifically observed in patients younger than 5 years old [3]. These findings prompted us to propose that EBV associated lymphomas in children from Argentina could be triggered as a complication from primary infection and, in consequence, to look forward for viral features that could possibly be related to these observations. Finally, the fact that only one EBV variant is usually detected in samples from pediatric patients with IM [42–44], allows us to identify the original infective variant, in contrast to what is frequently reported in adult patients where multiple variants may be detected due to subsequent re-infections during adolescence and early adulthood.

Regarding LMP1 gene, a classification system to define its sequence variation, based on signature polymorphisms in the C-ter region was introduced by Edwards et al. in the year 1999 (21) and is still the most frequently used system until today [23]. But, since geographical restrictions have been proposed for LMP1 variants and that it is plausible that a portion of any given gene may not reflect its total degree of variation, we assessed its variability, molecular evolution, disease and geographic association in 65 full-length LMP1 sequences from pediatric patients with benign or malignant EBV diseases and healthy carriers. A novel local variant, present in 46% of the total isolates was phylogenetically and molecularly characterized as the Argentine variant. The high prevalence of this variant in our region could reflect features such as geographically restrained distribution, genetics and/or immunological factors of the host. Moreover, these observations are strengthened by the fact that Zuercher et al. previously described the two signature polymorphisms (I124V/I152L) in an HIV cohort, but in a very low proportion of isolates and did not describe any recombination events [36]. The analysis of the entire LMP1 coding sequence was proven as a better strategy than only the C-ter region, even if the highest amount of polymorphisms are harbored within the latter region. In our previous work, when analyzing exclusively the C-ter region of LMP1 in different compartments in children with IM and pediatric EBV-related lymphomas from Argentina, China1 seemed to be the most common variant in our region [38]. The current analysis of the complete coding sequence of LMP1 allowed us to re-classify these isolates as a distinctive group that clustered in proximity to China1, but harbored significant differences, which made it more closely related to Raji variant. Indeed, the polymorphic hallmarks of Argentine variant were located between the N-ter and TM domains; hence the importance of analyzing full length sequences in order to define LMP1 variants. None the less, it was within the C-ter domain, where a recombination point, another hallmark of the Argentine variant, was located.

Most EBV associated tumors and EBV+ cell lines contain monoclonal EBV genomes [45]. On the other hand, co-infection with several EBV variants was reported in adult patients with IM [46–48], probably given by a higher viral inoculum transmitted through kissing; however, in pediatric primary infection these co-infections are infrequent, as observed in our previous reports in other EBV genes [38,42,49] and by others [43,44]. Indeed, when studying chronic active EBV infection in children, Jin et al. found a small proportion of co-infections but failed to do so in IM samples [44]. We hypothesize that this high EBV dose may include multiple variants which will give rise to co-infection in the adult host. On the contrary, the magnitude of viral dose acquired by a child through salivary contact, mediated by unclean toys or fingers, is lower, so the chances of being infected by multiple viral variants are less frequent. Besides there are only few studies about EBV variants in EBV+ reactive hyperplasia, where co-infections are detected in a small number of tonsils [50]. In our study, only one LMP1 variant was detected per sample which meant that during pediatric primary infection (IM patients), no differences were observed between anatomical compartments. This was also in accordance with our prior observations in our pediatric cohort when studying other EBV genes [38,42]. Moreover, no significant association between a given LMP1 variant and a certain pathological condition was discerned, since they were all similarly distributed between both benign and malignant conditions.

Concerning specific polymorphisms, XhoI-loss was the predominant variant detected in NPC by Nguyen-Van D et al. and Lin SX et al. so the authors speculated that this mutation could be associated with nasopharyngeal carcinogenesis [34,35]; however, in the present series this polymorphism was evenly observed in lymphomas, IM and RLH suggesting no involvement in lymphomagenesis. In accordance with the low incidence of XhoI-loss observed in our series, a recent review by Neves et al. highlights that even though associated to NPC development, this polymorphism could also be a geographic or race associated variation in South Asian population [23]. Whichever the case, XhoI-loss seems not be related to lymphomagenesis in South America nor a predominant polymorphism in our region.

Additionally, amino acid substitutions in the TM domain, particularly in those involved in LMP1 signaling activity, were proved to increase NF-kB activation in vitro and were also described as markers related to malignant processes [36]. However, in our pediatric series the combination of polymorphisms I124V/I152L and F144I/D150A/L151I within the TM domains were not associated with EBV related lymphomas. Concerning the C-ter of LMP1, different polymorphisms have been profusely described (20). A 30bp deletion has been associated with a variety of EBV-related tumors by several groups [20,51,52]. Instead, some others suggested that the high prevalence of the deleted-LMP1 variant represents a geographical or race-associated polymorphism rather than a disease-associated polymorphism (reviewed in [19,20]). As was previously reported for Argentina [38], the 30bp deletion was evenly detected among all the studied groups, further suggesting that it is not involved in lymphomagenesis. On the other hand, an insertion of 15bp (ins15) and a variable number of repetitive units of 33bp (rep33) were previously described in relation to tumorigenesis [38]. In our studied group, ins15 was predominantly detected in the fourth unit of rep33, but still proved not to be statistically associated with lymphomas. Finally, 5½ repetitive units of rep33 were the most frequently detected number of repetitions but still significantly present in a higher proportion of tumors than in healthy carriers, suggesting that this polymorphism could be related to the lymphomagenesis process.

EBV can also be classified into type I and II based on sequence differences in EBNA genes. While type 1 is worldwide distributed, type 2 is predominant in Equatorial Africa and Southeast Asia, [reviewed in [19,20]]. Based on this geographical distribution, previous studies also suggested a geographical association of LMP1 variants from Southeast Asia, Papua, New Guinea, Africa, and Australia [53]. The phylogeographic analysis performed in this study, which included isolates from different locations, proved that some of the LMP1 variants have a global distribution, while others, like the Asian or Argentinean variants, were restricted to a particular geographic region. Moreover, the Argentine variant appeared to be an admixture between African and Asian viral variants. All these results are in accordance with a recent report by Chiara et al., who also described the clustering of “pure” sub-populations in different geographic regions, namely Asia and Europe, but also described admixed viral populations between American and African variants [54]. The reasons for the restricted geographic pattern of EBV-associated entities in different regions are poorly understood, but variation in host genetics, such as HLA polymorphisms in different populations, or environmental factors, such as diet, could be speculated to account for geographically associated variations in the viral genome [20,54].

The estimated evolutionary rate for LMP1 by MCMC analysis, 8.591x10^-5^ subs/site/year over a period of 42 years, was higher than expected under the hypothesis of co-evolution with the host species, as compared to that for α-herpesvirus, which is estimated from 1x10^-7^ to 1x10^-9^ subs/site/year [55–57]. Additionally, 8 positively selected codons were detected in our isolates (data not shown) which could account for this augmented mutation rate; however, it should also be noted that this evolution rate applies only to the present dataset and does not necessarily apply to the rest of the EBV genome. In line with this, EBV latency genes were described to show greater diversity and faster evolution rates than lytic genes [16]. Moreover, the assumption that all DNA viruses evolve slower than RNA viruses, has been recently challenged [58–60]. In fact, by making use of this analysis Firth et al. estimated evolutionary parameters for double stranded DNA virus and concluded that given a dataset containing a large enough number of variable sites, it is possible to accurately estimate substitution rates that range from 10^−4^ to approximately 10^−7^ subs/site/year using temporally sampled viruses [58]. The authors suggested that two general mechanisms may influence the substitution rate of rapid evolving DNA viruses. First, as the rate of viral replication directly impacts in the long-term substitution rate, very high replication rates might increase the substitution rates of dsDNA viruses. Second, positive selection could also play a role in increasing the substitution rate over the expected mutation rate. Unfortunately, the replication rate of EBV during natural primary infections in the host is still unknown; however, the action of positive selection inferred from an increased proportion of non-synonymous variation has been already described in LMP1 and EBNA1 [39,53].

The tMRCA estimated for Raji and Argentine was 136 ybp (76–223 ybp, 95% HPD), which is coincident than the historical period of introduction of African slave into Argentina. In fact, during the 18-19^th^ centuries, 30% Buenos Aires citizens were of African origin, while in rural areas over 54% of the population were African slaves [61]. Moreover, the estimation is that 3–4% of the genetic ancestry of Buenos Aires City population is African [61]. It is then highly plausible that the LMP1 Argentine variant could occur from a recombination event between an ancestral worldwide distributed variant (China1) and an African one (Raji), following the introduction of African slaves.

In conclusion, our results highlight on the extent of EBV diversity, particularly in LMP1 gene which shows a complex evolutionary pattern that involves both selection and recombination processes. Furthermore, we characterized a predominant LMP1 variant potentially originated through recombination due to African migrations into our country.

In this way this work contributes to enlarge our present knowledge on structural variants or mutations from a still underrepresented geographic location [17,20,23], South America, which displays a characteristic epidemiology concerning EBV related diseases.

## Supporting information

S1 Fig Positional entropy. Shannon information entropy values for full length LMP1 alignment from patients with IM, EBV positive lymphomas and RLH, were plotted according to values generated in BioEdit. Amino acid positions that do not exhibit any changes have entropy of 0, whereas positions of high variability are represented by peak in the plot.
(TIF)

S2 Fig Randomization test. The first data point represents the substitution rate estimated for the entire set of data (grey circle) whereas the remaining 20 points (black circles) represent estimated rates with random isolation dates. Y axis is represented in a log_10_ scale.
(TIF)

S1 Table Patient’s isolates description. (DOC)

S2 Table Evolution model parameters for the different phylogenetic reconstructions as estimated with jModelTest. (DOCX)

S3 Table Results of Bayesian tips-significance tests (BaTS). Global association test between trait and tree topology.
(DOC)

S4 Table Mean and 95% HPD of the Bayesian Posterior Estimates of Substitution Rate (subs/site/year) and tMRCA. (DOC)
