# Supplementary figures and images for: A novel recombinant variant of latent membrane protein 1 from Epstein Barr virus in Argentina denotes phylogeographical association
Source: PLoS One. 2017 Mar 22;12(3):e0174221. doi: 10.1371/journal.pone.0174221 (PMC5362222; doi:10.1371/journal.pone.0174221)

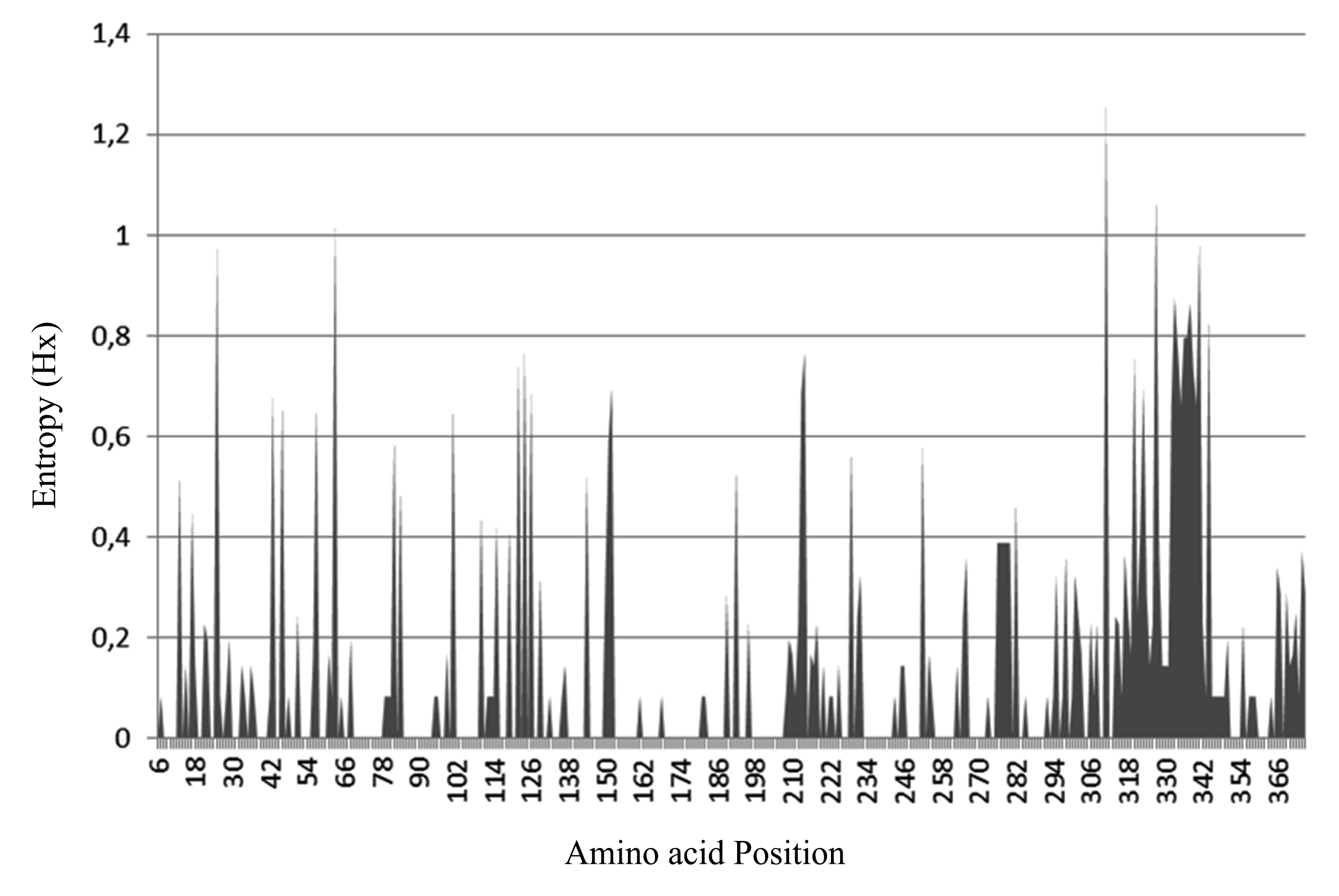

Supplement: S1 Fig — Shannon information entropy values for full length LMP1 alignment from patients with IM, EBV positive lymphomas and RLH, were plotted according to values generated in BioEdit. Amino acid positions that do not exhibit any changes have entropy of 0, whereas positions of high variability are represented by peak in the plot. (TIF) [file pone.0174221.s001.tif]

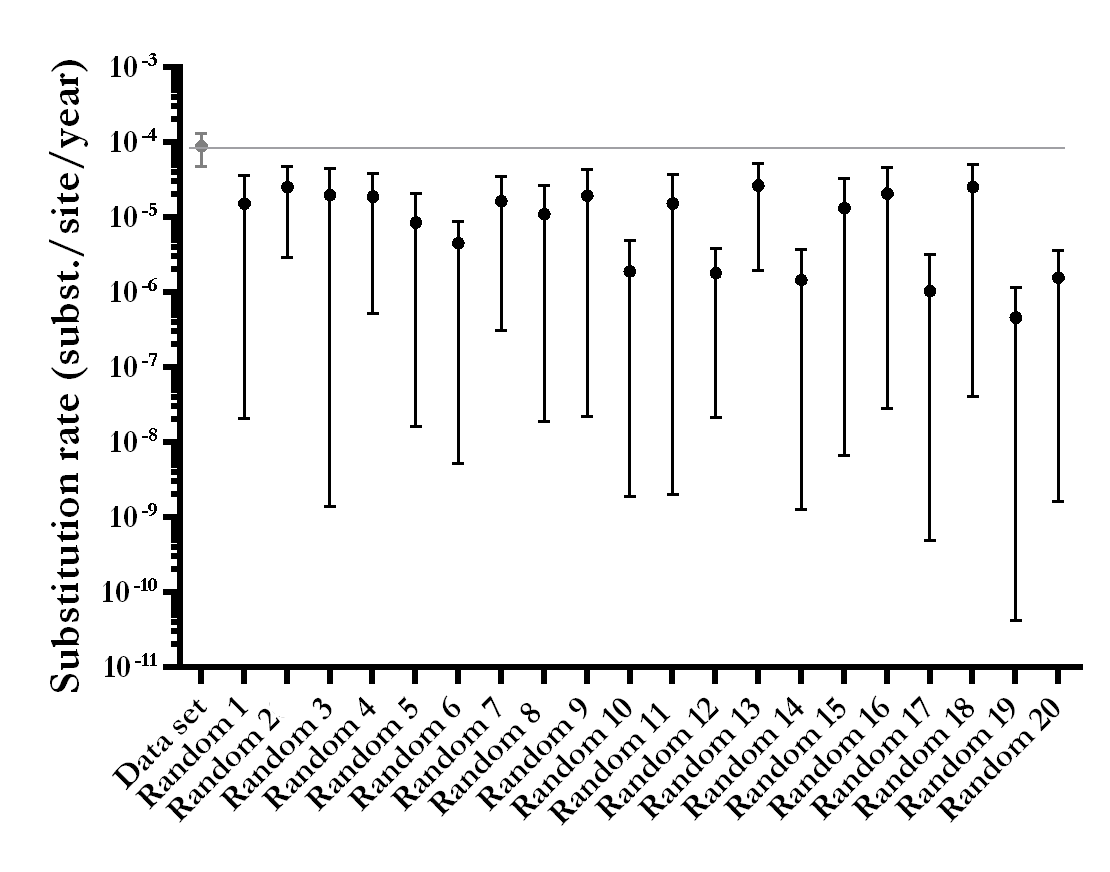

Supplement: S2 Fig — The first data point represents the substitution rate estimated for the entire set of data (grey circle) whereas the remaining 20 points (black circles) represent estimated rates with random isolation dates. Y axis is represented in a log10 scale. (TIF) [file pone.0174221.s002.tif]
