# Supplementary material for: A novel recombinant variant of latent membrane protein 1 from Epstein Barr virus in Argentina denotes phylogeographical association
Source: PLoS One. 2017 Mar 22;12(3):e0174221. doi: 10.1371/journal.pone.0174221 (PMC5362222; doi:10.1371/journal.pone.0174221)
Supplement: S1 Table — (DOC) [file pone.0174221.s003.doc]

**S1 Table. Patient’s isolates description.**

| **Isolate** | **Age** | **Sex** | **Diagnosis** | **IgG VCA** | **Isolation date** | **EBV type** |
| --- | --- | --- | --- | --- | --- | --- |
| IM1 | 9 | M | IM | + | 2007 | I |
| IM2 | 2.5 | M | IM | + | 2007 | I |
| IM3 | 3 | M | IM | + | 2007 | I |
| IM4 | 2 | M | IM | + | 2007 | I |
| IM5 | 4 | F | IM | + | 2008 | II |
| IM6 | 3 | F | IM | + | 2008 | I |
| IM7 | 4.5 | M | IM | + | 2008 | II |
| IM8 | 5 | F | IM | + | 2008 | I |
| IM9 | 6 | F | IM | + | 2008 | I |
| IM10 | 8 | F | IM | + | 2008 | I |
| IM11 | 2.5 | M | IM | + | 2008 | I |
| IM12 | 17 | F | IM | + | 2010 | I |
| IM13 | 1.5 | F | IM | + | 2010 | I |
| IM14 | 6 | F | IM | + | 2010 | I |
| IM15 | 8.5 | M | IM | + | 2011 | I |
| IM16 | 6 | M | IM | + | 2011 | I |
| IM17 | 3 | F | IM | + | 2011 | I |
| IM18 | 6 | M | IM | + | 2011 | I |
| IM19 | 2 | M | IM | + | 2012 | I |
| IM20 | 3 | F | IM | + | 2012 | I |
| IM21 | 7 | F | IM | + | 2012 | I |
| IM22 | 3.5 | M | IM | + | 2013 | I |
| IM23 | 4 | M | IM | + | 2013 | I |
| IM24 | 5 | F | IM | + | 2013 | I |
| IM26 | 6 | M | IM | + | 2013 | I |
| IM27 | 3 | M | IM | + | 2014 | II |
| IM28 | 7 | M | IM | + | 2014 | II |
| IM29 | 8 | M | IM | + | 2014 | I |
| IM30 | 2.5 | F | IM | + | 2015 | I |
| IM31 | 4.5 | F | IM | + | 2015 | I |
| **Isolate** | **Age** | **Sex** | **Diagnosis** | **EBERs** | **Isolation date** | **EBV type** |
| T1 | 14 | M | DLBCL | + | 2000 | II |
| T2 | 9 | M | DLBCL | + | 1995 | II |
| T3 | 7 | F | DLBCL | + | 2001 | I |
| T4 | 5 | M | HL | + | 2003 | I |
| T5 | 5 | M | HL | + | 2000 | I |
| T6 | 12 | F | HL | + | 2003 | II |
| T7 | 6 | M | HL | + | 1999 | I |
| T8 | 15 | F | HL | + | 1996 | II |
| T9 | 6 | M | HL | + | 2003 | I |
| T10 | 10 | M | HL | + | 2001 | II |
| T11 | 9 | M | HL | + | 2006 | I |
| T14 | 7 | M | HL | + | 1994 | I |
| T15 | 8 | M | ALCL | + | 1995 | I |
| T16 | 8 | M | HL | + | 1998 | II |
| T18 | 6 | F | DLBCL | + | 1993 | I |
| T19 | 4 | F | HL | + | 2009 | I |
| T21 | 7 | M | HL | + | 2007 | I |
| T22 | 10 | M | BL | + | 2009 | I |
| T23 | 8 | M | HL | + | 2011 | II |
| T24 | 8 | M | HL | + | 2011 | I |
| T25 | 10 | M | HL | + | 2011 | II |
| T26 | 16 | M | HL | + | 2010 | I |
| T27 | 9 | M | HL | + | 2010 | I |
| T28 | 6 | M | HL | + | 2010 | I |
| T29 | 17 | M | HL | + | 2010 | I |
| T30 | 10 | M | HL | + | 2011 | I |
| T31 | 13 | F | HL | + | 2013 | I |
| T32 | 9 | M | HL | + | 2013 | II |
| T33 | 18 | M | HL | + | 2014 | II |
| **Isolate** | **Age** | **Sex** | **Diagnosis** | **EBERs** | **Isolation date** | **EBV type** |
| RLH1 | 9 | F | RLH | + | 2012 | I |
| RLH2 | 5 | F | RLH | + | 2003 | II |
| RLH3 | 3 | M | RLH | + | 2003 | I |
| RLH4 | 9 | M | RLH | + | 2003 | II |
| RLH5 | 11 | M | RLH | + | 2003 | II |
| RLH6 | 5 | F | RLH | + | 2003 | II |
| RLH7 | 7 | F | RLH | + | 2012 | II |
| RLH8 | 8 | M | RLH | + | 2001 | I |
| RLH9 | 16 | F | RLH | + | 2003 | I |
| RLH10 | 3 | M | RLH | + | 2002 | I |
| RLH11 | 8 | F | RLH | + | 2009 | I |
| RLH12 | 12 | M | RLH | + | 2005 | I |
| RLH13 | 9 | M | RLH | + | 2008 | I |
| RLH14 | 7 | M | RLH | + | 2011 | I |

IM: Infectious mononucleosis; HL: Hodgkin lymphoma; DLBCL: Diffuse large B cell lymphoma; ALTCL: Anaplastic large cell lymphoma; BL: Burkitt lymphoma; RLH: Reactive lymphoid hyperplasia; M: Male; F: Female.
