# Supplementary material for: A novel recombinant variant of latent membrane protein 1 from Epstein Barr virus in Argentina denotes phylogeographical association
Source: PLoS One. 2017 Mar 22;12(3):e0174221. doi: 10.1371/journal.pone.0174221 (PMC5362222; doi:10.1371/journal.pone.0174221)
Supplement: S2 Table — (DOCX) [file pone.0174221.s004.docx]

| Alignment | LMP1 Complete (Fig. 1) | LMP1  N-Ter + TM (Fig. 2A) | LMP1 C-Ter (Fig. 2B) | Phylogeographic analysis (Fig. 5) |
| --- | --- | --- | --- | --- |
| Selected model | 012033+I+G | 012344+I+G | TIM3 012032+I+G | GTR |
| (Partition code) |  |  |  | 012345+I+G |
| Base Frequency |  |  |  |  |
| A | 0.2056 | 0.1627 | 0.2468 | 0.1904 |
| C | 0.3275 | 0.3213 | 0.3394 | 0.3360 |
| G | 0.1963 | 0.1745 | 0.2377 | 0,211 |
| T | 0.2706 | 0.3415 | 0.1762 | 0.2621 |
| Substitution rates |  |  |  |  |
| A-C | 2.0963 | 3.2712 | 1.4574 | 2.2183 |
| A-G | 3.9679 | 4.7207 | 2.8500 | 4.4907 |
| A-T | 0.6138 | 0.5598 | 1 | 0.7498 |
| C-G | 2.0963 | 2.0364 | 1.4574 | 1.7996 |
| C-T | 1 | 1 | 0.8906 | 1.1100 |
| G-T | 1 | 1 | 1 | 1 |
| I | 0.4550 | 0.3930 | 0.3850 | 0.2740 |
| G | 0.9240 | 1.0500 | 0.8880 | 0.8090 |

**S2 Table. Evolution model parameters for the different phylogenetic reconstructions as estimated with jModelTest.**
