# Supplementary material for: A novel recombinant variant of latent membrane protein 1 from Epstein Barr virus in Argentina denotes phylogeographical association
Source: PLoS One. 2017 Mar 22;12(3):e0174221. doi: 10.1371/journal.pone.0174221 (PMC5362222; doi:10.1371/journal.pone.0174221)
Supplement: S3 Table — Global association test between trait and tree topology. (DOC) [file pone.0174221.s005.doc]

**S3 Table**. **Results of Bayesian tips-significance tests (BaTS). Global association test between trait and tree topology.**

| **Statistic** | **Observed mean (95% Ci)** | **Null mean (95% Ci)** | **Significance level** |
| --- | --- | --- | --- |
|
| AI | 10.1 (8.98-11.23) | 22.5 (21.5-24.2) | <0.001 |
| PS | 76.4 (73-79) | 145 (140.3-151.2) | <0.001 |
| MC (Argentina) | 5.9 (4-10) | 2.7 (2.3-3.4) | 0.004 |
| MC (Asia) | 9.92 (6-17) | 2.52 (2-3,2) | <0.001 |
| MC (Europe) | 2.7 (2-4) | 2.2 (1,72-3) | 0.08 |
| MC (Africa) | 10.8 (9-12) | 1.9 (1.3-2.9) | <0.001 |
| MC (North America) | 2.9 (2.7-3) | 1.32 (1-2) | 0.001 |
| MC (Oceania) | 2.8 (2-4) | 1.48 (1-2) | 0.002 |

AI: Association Index; PS: Parsimony store; MC: Monophiletic clade
