# Supplementary material for: A novel recombinant variant of latent membrane protein 1 from Epstein Barr virus in Argentina denotes phylogeographical association
Source: PLoS One. 2017 Mar 22;12(3):e0174221. doi: 10.1371/journal.pone.0174221 (PMC5362222; doi:10.1371/journal.pone.0174221)
Supplement: S4 Table — (DOC) [file pone.0174221.s006.doc]

S4 Table. Mean and 95% HPD of the Bayesian Posterior Estimates of Substitution Rate (subs/site/year) and tMRCA.

| **Molecular clock model** | **Demographic model** | **Marginal Likelihood** | **Substitution rate (subst./site/ year)** | **Substitution rate** **95% HPD** | **tMRCA** | **tACMR 95% HPD** |
| --- | --- | --- | --- | --- | --- | --- |
| Strict | Constant | -5661.164 | 8.246 x 10^-5^ | 4.001-12.25 x 10^-5^ | 402 | 215-647 |
|  | Exponential | -5660.164 | 7.512 x 10^-5^ | 3.198-17.22 x 10^-5^ | 430 | 214-2357 |
|  | BS | -5662.558 | 8.077 x 10^-5^ | 3.454-12.36 x 10^-5^ | 405 | 205-695 |
| UCLN | Constant* | -5657.725 | 8.591 x 10^-5^ | 4.779-12.89 x 10^-5^ | 393 | 216-608 |
|  | Exponential | -5658.331 | 7.935 x 10^-5^ | 3.393-12.24 x 10^-5^ | 411 | 205-706 |
|  | BS | -5659.544 | 8.47 x 10^-5^ | 3.770-13.33 x 10^-5^ | 395 | 194-684 |

* Denotes the method which maximizes the posterior probability (Bayes Factor)
